# Wellens' Syndrome: A Sign of Impending Myocardial Infarction

**DOI:** 10.7759/cureus.26084

**Published:** 2022-06-19

**Authors:** Reshmi Mathew, Yixin Zhang, Christopher Izzo, Pramod Reddy

**Affiliations:** 1 Internal Medicine, University of Florida College of Medicine – Jacksonville, Jacksonville , USA; 2 Internal Medicine, University of Florida College of Medicine – Jacksonville, Jacksonville, USA

**Keywords:** left anterior descending artery (lad), myocardial infarction, pseudo-wellens, left ventricular hypertrophy (lvh), wellens’, wellens’ syndrome

## Abstract

Wellens’ pattern is an electrocardiogram (EKG) finding of biphasic or deeply inverted T waves in leads V2 and V3 that is suggestive of anterior wall ischemia classically reflecting critical stenosis of the proximal left anterior descending artery (LAD). This pattern reflects a preinfarction state that can eventually progress to massive and fatal anterior wall myocardial infarction (MI). We describe a case of a 50-year-old male who presented with chest pain and hypertensive emergency. EKG revealed new biphasic T-waves in V2-V5. The patient's chest pain resolved with blood pressure control, however, the persistence of Wellens' pattern on EKG prompted further investigation. Emergent left heart catheterization (LHC) revealed severe multivessel coronary artery disease, most notably with critical stenosis of the mid-LAD. The patient underwent successful surgical revascularization. This case highlights a classic EKG pattern that can have serious morbidity and mortality if it is missed. This case also describes a unique anatomical correlation of Wellens' syndrome as the coronary lesion was identified in the mid-LAD, contrary to lesions typically identified in the proximal LAD. Prior knowledge about Wellens’ pattern allowed us to consider the possibility of critical LAD stenosis, which allowed for timely intervention and prevention of a massive myocardial infarction and possibly death.

## Introduction

Wellens’ pattern is an EKG finding of biphasic or deeply inverted T waves in leads V2 and V3 that is indicative of critical stenosis of the left anterior descending artery (LAD) [1]. There are two recognized Wellens’ patterns, type A and type B [1]. Type A includes the presence of biphasic T waves in V2 and V3 [1]. Type B includes inverted T waves in leads V2 and V3 [1]. These patterns often reflect a preinfarction state [1], making early recognition and treatment imperative. This diagnosis can easily be missed due to patients often presenting in a pain-free state, with only subtle EKG changes and little to no rise in cardiac biomarkers, a combination of which can be falsely reassuring. After accounting for cardiac risk factors and the presence of Wellens' pattern on EKG, coronary evaluation is imperative [1]. The decision for medical management versus surgical management is then tailored based on coronary disease burden. We describe a patient who presented with chest pain and whose EKG revealed Wellens' pattern. Recognition of the persistence of Wellens’ pattern on serial EKGs prompted the decision for left heart catheterization (LHC) and early revascularization. This case attempts to bring increased awareness of a classic EKG finding and its angiographic correlation to prevent serious morbidity and mortality in patients who have variable clinical presentations.

## Case presentation

**Figure 1:**
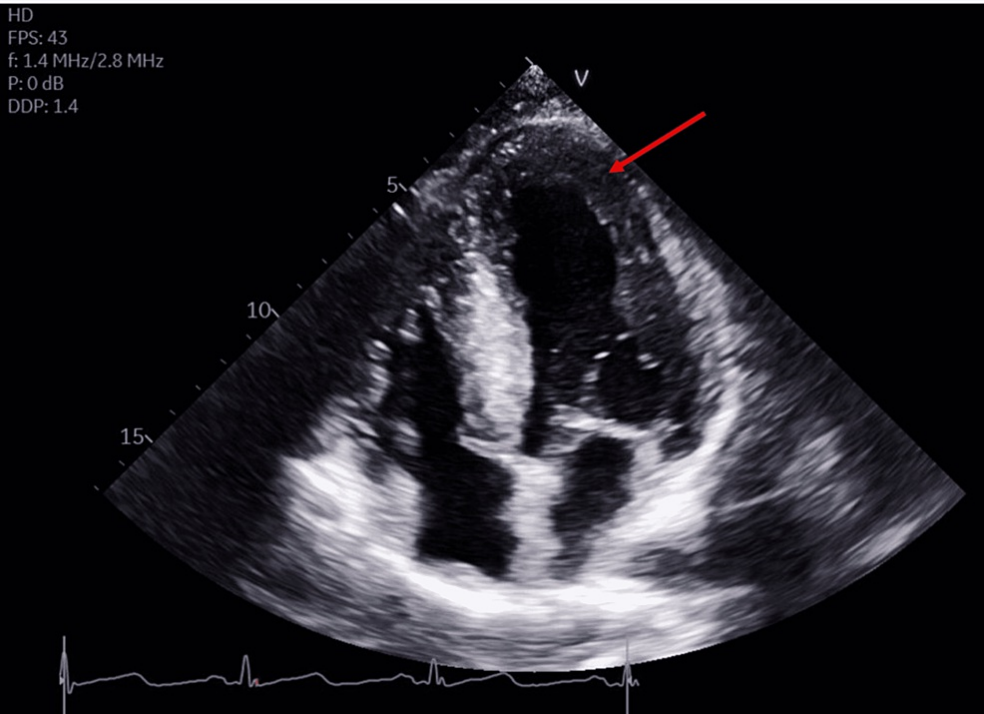
Transthoracic echocardiogram Transthoracic echocardiogram (TTE) in apical four-chamber view shows severe concentric left ventricular hypertrophy (LVH) (arrow). The ejection fraction was 60-65%. All segments contract normally. The diastolic filling pattern indicates impaired relaxation and elevated left ventricular end-diastolic pressure.

A 50-year-old male presented with acute onset chest pressure associated with shortness of breath, palpitations, and lightheadedness. His chest pain began five hours prior to the presentation while he was doing exertional yard work. The patient’s past medical history includes hypertension, hyperlipidemia, tobacco, cocaine, and marijuana use with medical noncompliance. The pain was characterized as sudden-onset, severe substernal chest pain that lasted about 20 seconds and subsided with rest. He also reported prior episodes of exertional chest pain relieved by rest in the weeks preceding presentation. The patient reported noncompliance with his home antihypertensive medications. The patient was an active tobacco smoker with a 15-pack-year smoking history. On presentation to the emergency department, the patient was hypertensive with a blood pressure of 232/131 mmHg. All other vital signs were stable. Comprehensive metabolic panel and complete blood count were unremarkable. EKG on admission had evidence of dynamic T wave changes, including biphasic T waves in V2, V3, V4, and V5. Serial conventional troponin T assays at 0, 6, and 12 hours from the presentation were negative. Transthoracic echocardiogram (TTE) showed evidence of severe concentric left ventricular hypertrophy (LVH) with preserved ejection fraction (Figure 1).

**Figure 2:**
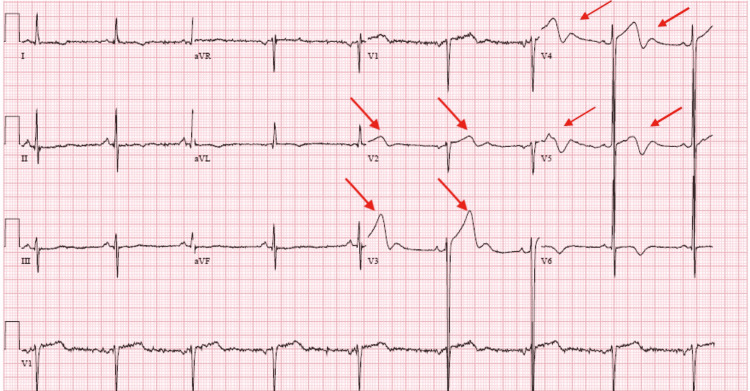
EKG on arrival EKG on arrival showed Type A Wellens' pattern with biphasic T waves in V2-V5. Additionally, there are T wave inversions in leads I and aVL. There is LVH by Cornell criteria and J point elevation with upsloping ST segments from V2-V5. LVH: Left ventricular hypertrophy

The urine drug screen was negative. On physical examination, the patient had normal S1 and S2 with no murmurs. The patient was treated with aspirin, statin, heparin infusion, sublingual nitroglycerin, and antihypertensive medications with a resolution of chest pain. However, repeat EKG showed persistent biphasic T waves in V2-V5 (Figure 2). 

**Figure 3:**
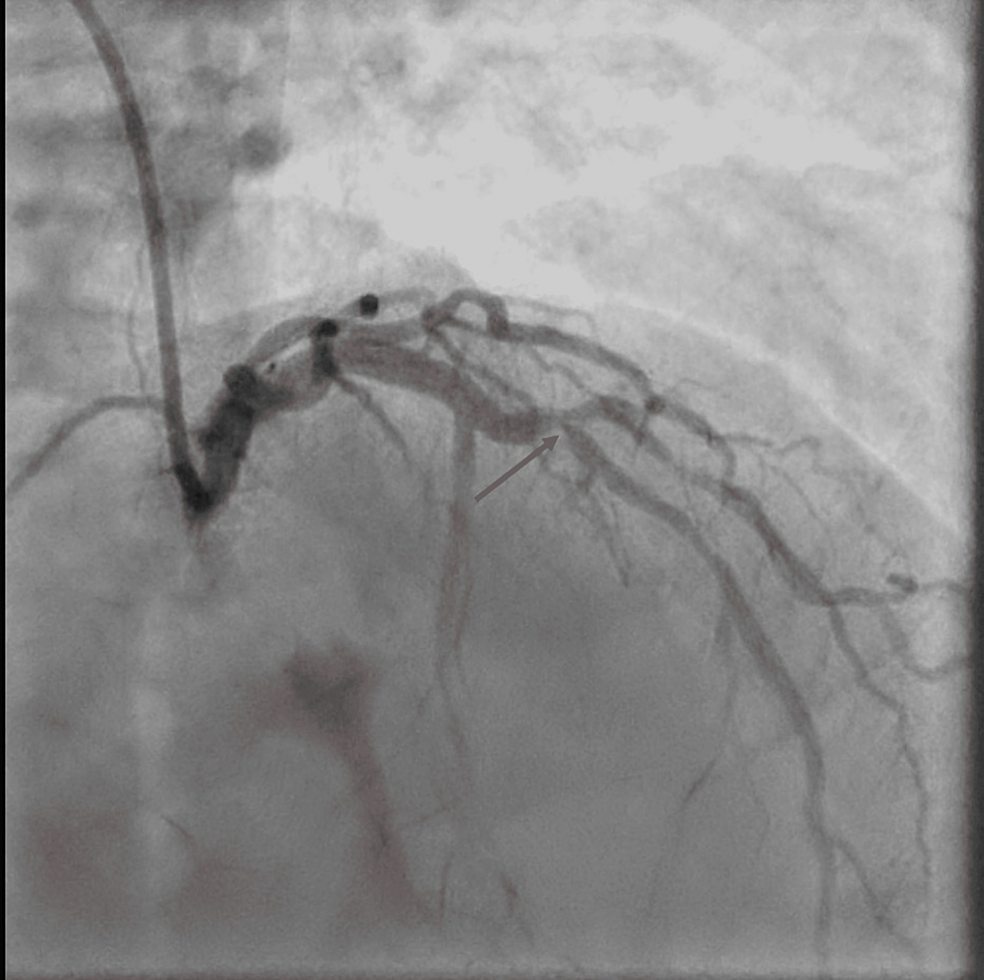
Left coronary angiogram in right anterior oblique cranial projection Left coronary angiogram in right anterior oblique cranial projection showing critical stenosis of the mid-LAD at the bifurcation of the first diagonal branch (arrow). LAD: Left anterior descending artery

GRACE (Global Registry of Acute Coronary Events) acute coronary syndrome (ACS) risk score was suggestive of low risk. However, due to concerns for Wellens’ syndrome on presenting EKG, the patient underwent coronary evaluation with LHC. Findings of LHC revealed severe multivessel coronary artery disease, most notably with 90% stenosis of the mid-LAD at the bifurcation of the first diagonal branch (Figure 3), followed by 80% tubular stenosis just distal to the D1 branch.

**Figure 4:**
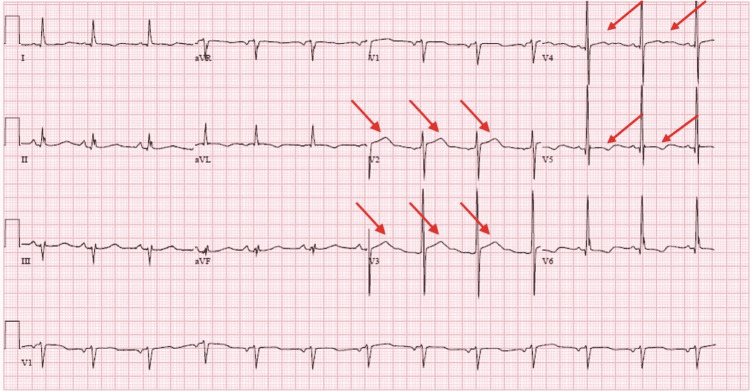
EKG after CABG showed resolution of biphasic T waves in septal leads. CABG: Coronary artery bypass graft

The right coronary artery had evidence of ostial 50-60% focal stenosis and 70-80% proximal stenosis. There was focal 80% distal stenosis at the bifurcation of the right-posterior descending artery and right-posterior left ventricular artery. Given the extent of his coronary artery disease, the patient underwent successful surgical revascularization with a 4-vessel coronary artery bypass graft (CABG). EKG performed postoperatively showed complete resolution of biphasic T waves in V2, V3, V4, and V5 (Figure 4). 

The patient tolerated the procedure well and was discharged home six days after surgery with outpatient cardiology follow-up. 

## Discussion

Wellens’ syndrome was first recognized by Dr. Zwaan and colleagues in the 1980s. Wellens’ syndrome is characterized by EKG findings of biphasic or deeply inverted T waves in V2 and V3. Uncommonly, the T wave changes may also extend into leads V1, V4, V5, and V6 [1], as it did with our patient. One study found that T-wave inversions (TWIs) signifying critical LAD stenosis had a sensitivity of 69%, specificity of 89%, and a positive predictive value of 86% [2]. The diagnosis also requires that there is a history of angina, less than 1 mm ST-segment elevation throughout, preservation of R-wave progression, and no precordial Q waves [1].

There are two basic patterns of T wave changes seen in Wellens’ syndrome. Type A pattern includes biphasic T waves in V2-V3, which are initially positive and terminally negative [3]. Type A pattern is present in approximately 25% of cases [1]. Type B pattern includes deeply inverted T waves in V2-V3 and occurs in about 75% of cases [1,3]. These two changes often exist on a spectrum that progresses from type A to type B with the progression of the disease [3]. It is important to note that these EKG abnormalities may persist even when patients are in a pain-free state, as occurred with our patient. The TWIs in Wellens’ syndrome have been reported to persist on EKG for hours to even weeks [4].

Patients with Wellens’ syndrome are often difficult to identify due to subtle changes on EKG and minimal to no increase in cardiac biomarker levels. Most patients with Wellens’ syndrome also present in a pain-free state, resulting in low clinical suspicion for ACS. Wellens’ syndrome has been postulated to occur due to rupture of an atherosclerotic plaque that then leads to transient occlusion of the LAD, with probable clot lysis of the occlusion before myocardial infarction occurs [1]. 

Although TWIs in the anterior leads on EKG can be indicative of Wellens’ pattern, the differential diagnoses for TWI remain numerous. TWI in the anterior leads can appear on EKG due to LVH, bundle branch block, pulmonary embolism, and digitalis or cocaine-induced coronary vasospasm [5]. Our patient had severe concentric LVH on TTE. Additionally, he presented in a state of hypertensive emergency. It is possible for ST-T segment changes to be seen in LVH due to repolarization abnormalities of the hypertrophied muscle of the left ventricle [6]. Additionally, the dynamic T-wave changes on EKG could have been attributed to demand ischemia in the setting of supply-demand mismatch that can be seen in hypertensive emergencies. It was the persistence of EKG changes after blood pressure control and resolution of chest pain that increased suspicion of underlying coronary artery disease. 

The risk factors for Wellens’ syndrome are similar to other conditions that cause cardiac disease, including hypertension, diabetes, hyperlipidemia, tobacco use, and increased age. Obtaining a thorough history is also important because there are a few factors that can cause pseudo-Wellens’ syndrome. Patients with pseudo-Wellens' syndrome have the characteristic Wellens’ pattern on EKG, but they will have normal coronary anatomy on further investigation [7]. Pseudo-Wellens’ syndrome has been associated with cocaine, heavy marijuana use, acute cholecystitis, and Takotsubo cardiomyopathy [7].

Wellens’ syndrome is treated similar to how one would treat an acute myocardial infarction. Patients with suspicious EKG findings should be started on antiplatelet therapy with aspirin, anticoagulation with heparin, beta-blockers, and nitrates [1]. However, it is important to note that definitive management includes coronary angiography and revascularization [1]. Dr. Zwaan and colleagues showed that patients with Wellens’ syndrome who were treated with medical therapy alone developed myocardial infarction in the ensuing weeks [8]. This makes revascularization of paramount importance to prevent morbidity and mortality. Patients with multivessel coronary artery disease have better survival rates, lower rates of major cardiovascular events and repeat vascularization when undergoing CABG as compared with those having percutaneous coronary intervention (PCI) with drug-eluting stents [9]. The extent of coronary artery disease made our patient a better candidate for revascularization with CABG as compared with PCI. 

Timely recognition of this pattern is also imperative to avoid interventions that could have fatal outcomes. Specifically, stress tests are contraindicated in patients with Wellens' syndrome [10]. A stress test in patients with Wellens’ pattern on EKG will cause increased oxygen demand that can precipitate acute myocardial infarction, arrhythmias and even sudden death [1]. Inappropriate diagnostic workups and fatal outcomes can be avoided if physicians are able to quickly identify the subtle EKG findings present in Wellens’ syndrome.

## Conclusions

Wellens’ syndrome signifies critical stenosis of the LAD and can be indicative of impending anterior wall myocardial infarction. Patients typically present with no active chest pain and minimal to no rise in cardiac biomarkers, which can be falsely reassuring. This makes Wellens’ syndrome an easily overlooked diagnosis that can have fatal outcomes if missed. Emergency room physicians and internal medicine physicians are typically the first to encounter these initial EKGs on a patient’s presentation to the hospital. Therefore, there must be increased awareness of this diagnosis for early identification and prevention of serious morbidity and mortality. Although medical management is recommended initially, definitive management must be done with coronary evaluation. Physicians who promptly recognize this EKG pattern can advocate for patients to undergo definitive therapy with coronary angiography and revascularization. Our patient had early recognition and intervention that enabled us to halt the progression to a large and potentially fatal anterior wall myocardial infarction.
